# Classification of long-term condition patterns in rheumatoid arthritis and associations with adverse health events: a UK Biobank cohort study

**DOI:** 10.1177/26335565221148616

**Published:** 2023-02-10

**Authors:** Philip McLoone, Bhautesh D Jani, Stefan Siebert, Fraser R Morton, Jordan Canning, Sara Macdonald, Frances S Mair, Barbara I Nicholl

**Affiliations:** 1 General Practice and Primary Care, School of Health and Wellbeing, 223860 University of Glasgow, UK; 2 School of Infection and Immunity, 223860 University of Glasgow, Glasgow, UK

**Keywords:** Rheumatoid arthritis, latent class analysis, multimorbidity, comorbidity, mortality

## Abstract

**Purpose:**

We aimed to classify individuals with RA and ≥2 additional long-term conditions (LTCs) and describe the association between different LTC classes, number of LTCs and adverse health outcomes.

**Methods:**

We used UK Biobank participants who reported RA (n=5,625) and employed latent class analysis (LCA) to create classes of LTC combinations for those with ≥2 additional LTCs. Cox-proportional hazard and negative binomial regression were used to compare the risk of all-cause mortality, major adverse cardiac events (MACE), and number of emergency hospitalisations over an 11-year follow-up across the different LTC classes and in those with RA plus one additional LTC. Persons with RA without LTCs were the reference group. Analyses were adjusted for demographic characteristics, smoking, BMI, alcohol consumption and physical activity.

**Results:**

A total of 2,566 (46%) participants reported ≥2 LTCs in addition to RA. This involved 1,138 distinct LTC combinations of which 86% were reported by ≤2 individuals. LCA identified 5 morbidity-classes. The distinctive condition in the class with the highest mortality was cancer (class 5; HR 2.66 95%CI (1.91-3.70)). The highest MACE (HR 2.95 95%CI (2.11-4.14)) and emergency hospitalisations (rate ratio 3.01 (2.56-3.54)) were observed in class 3 which comprised asthma, COPD & CHD. There was an increase in mortality, MACE and emergency hospital admissions within each class as the number of LTCs increased.

**Conclusions:**

The risk of adverse health outcomes in RA varied with different patterns of multimorbidity. The pattern of multimorbidity should be considered in risk assessment and formulating management plans in patients with RA.

## Introduction

RA is a chronic disease that is characterised by joint inflammation and pain.^1^ In the United Kingdom (UK), the prevalence has been estimated to be between 0.7 and 1%, with the highest incidence occurring between the ages of 70 and 79.^2,3^ The systemic inflammatory process involved in RA is associated with an increased risk of other long-term conditions (LTCs) such as hypertension,^4^ diabetes mellitus,^5^ stroke,^6^ cardiovascular disease,^7^ chronic obstructive pulmonary disease (COPD), renal disease, liver disease and cancer.^8–11^ The painful and debilitating nature of RA means that those with the condition can experience a reduced quality of life and significant mental health problems such as depression and anxiety.^12^ Treatment with disease-modifying anti-rheumatic drugs and glucocorticoids can reduce inflammation and improve quality of life, but they also carry a risk of iatrogenic disease.^13^

There is a growing awareness that individuals with two or more LTCs (defined as multimorbidity) can have complex health care needs. Although several studies^4–12^ have explored the increased risk of specific individual LTCs among people with RA, it is recognised that some people with RA will experience several concurrent LTCs. Research on the patterns of disease combinations among people with RA and multimorbidity and the associated complexity of health care need is limited.^14^

We have previously reported on the number of LTCs and adverse health outcomes among participants in UK Biobank who had self-reported RA.^15^ In that paper we showed that there was an increased risk of mortality with increasing LTC count. In this paper we extend this work to explore the combinations of LTCs in people with RA. Firstly, we describe the different combinations of LTCs in people with RA and use exploratory techniques to examine whether persons with RA and multimorbidity (≥2 LTCs) can be usefully categorised by employing latent class analysis (LCA).^16^ Secondly, for participants assigned to the classes derived using LCA, we describe the incidence of mortality, major cardiac events, and emergency hospitalisation over an 11-year follow-up.

## Materials and methods

### Data source and study design

The UK Biobank is a prospective population-based research cohort that enrolled participants aged 37 to 73 between 2006 and 2010.^17^ All participants provided written informed consent which allowed for long-term follow-up and permitted linkage to their hospital discharge and death records.

The UK Biobank self-reported health conditions protocol is based on a touch screen questionnaire and nurse-led interview. Our study involved 5,658 participants who self-reported RA, and we investigated the combinations of other LTCs that they also reported. We limited these other conditions to a list of 42 LTCs (Supplementary Table S1) which we have previously used to study multimorbidity in UK Biobank.^18,19^ Each LTC was defined as a binary variable (either present or absent). A total of 33 participants did not indicate whether they had ever received a cancer diagnosis. These participants were excluded leaving an analytical sample of 5,625 participants.

Baseline characteristics included sex, age (categorised as <50, 50-59, 60+ years), body mass index (BMI; grouped using WHO BMI categories^20^ underweight <18.5, recommended weight 18.5-<25, overweight 25-<30 and obese ≥30kg/m^2^), smoking status (never or current/ex-smokers), alcohol consumption frequency (never/special occasions, 1 to 3 times a month, at least once a week), physical activity (none, low, moderate, and high based on metabolic equivalent task scores^21^ derived from the short International Physical Activity Questionnaire), and residence-based Townsend socioeconomic deprivation scores^22^ grouped into quintiles of the distribution of scores in the UK Biobank sample. BMI, smoking status, alcohol consumption, physical activity and Townsend scores were not recorded for 53 (0.9%), 47 (0.8%), 10 (0.2%), 137 (2.2%) and 10 (0.2%) participants, respectively. Complete baseline characteristics were available for 5,407 (96.1%) of included participants.

### Outcomes

Follow-up for the occurrence of adverse health events began from date of baseline assessment and was measured by all-cause mortality, the number of emergency hospitalisations (defined by continuous in-patient stays derived from admission and discharge dates), and major adverse cardiac events (MACE). MACE was defined as hospitalisation with a primary diagnosis of stroke (ICD-10 I60-69) or myocardial infarction (I21-25) or mortality due to cardiovascular disease (I00-I78, G45 and G46). For all-cause mortality, follow-up ended on 28 February 2020. Follow-up for MACE was until date of MACE, date of death or 28 February 2020 (28 February 2018 for residents in Wales because this was the latest date that hospitalisation data were available) whichever occurred first. Follow-up for emergency hospitalisation was until date of death or 28 February 2020 (28 February 2018 for residents in Wales) whichever occurred first.

### Statistical methods

We described the association between LTCs using tetrachoric correlations (a measure of association between dichotomous variables). For participants who reported RA with ≥2 other LTCs we used latent class analysis (LCA; poLCA package R version 4.0.4) to classify participants into categories based on the presence or absence of each LTC. LCA is a model-based probabilistic approach to identifying categories of an unobserved latent variable.^15^ In this study the sets of LTCs that participants with multimorbidity reported were regarded as manifestations of the latent variable. This classification approach means that individuals within a class need not necessarily have the same combination of conditions, and each individual condition can feature in all classes. There will, however, be differences between classes in the prevalence of specific LTCs.

LCA was carried out using all participants in the analytical sample. Starting with two classes, we fitted all LCA models from two to 10 classes. Each model was repeated 500 times with 6,000 iterations to enable the identification of the global maximum likelihood and ensure convergence. The choice of number of classes to retain was guided by the characteristics of classes as their number increased and by observing a combination of statistics (sample size-adjusted Bayesian Information Criteria (BIC) and entropy for classification quality.)^23^ The BIC checks how well a model fits but penalises overfitting. Entropy summarises the probability of each person being in each class, higher values indicating more precise assignment. Classes were numbered in the order generated. For ease of reference, classes are referred to by (at most) three 'distinctive' LTCs that distinguished classes from one another. The distinctive conditions were ranked and defined as those LTCs which showed the largest difference in prevalence when compared to the prevalence in the overall sample.

To assess the stability of the different classes, the LCA was repeated using the 16 LTCs which had an overall prevalence of at least 2% in the study sample (Supplementary Table S2). Each class derived from 42 LTCs was matched with a corresponding class in the secondary LTC analysis. An overall measure of agreement between the two approaches was derived from the percentage of participants who appeared in equivalent classes in both models.

For each class we described the prevalence and combination of LTCs. To quantify the association between outcomes, multimorbidity classes, demographics, BMI and health-related behaviours, Cox proportional hazard models (using age as the time scale) were used for MACE and overall mortality, and negative binomial regression (with follow-up time as the exposure) was used for number of emergency hospitalisation admissions. These analyses initially adjusted for age and sex, and then took account of smoking, deprivation, BMI, alcohol consumption and physical activity. Individuals who reported RA with only one LTC were included as a separate ‘class’ in the analyses. Individuals with RA and no other LTCs were used as the reference group. We repeated the analysis sub-dividing classes by number of LTCs (defined as 2, 3 or ≥4). Regression models excluded participants with missing baseline data.

## Results

The median age of participants with RA was 62 (IQR 57-66) years and 70% were women. Just under one third were obese (32%), 53% were current or previous smokers and 25% resided in the most deprived quintile of area deprivation scores of UK Biobank participants (see Table 1).

**Table 1.** Participant characteristics. Numbers (percentages) of persons with RA with no additional long-term condition (LTC), with one additional LTC, and with ≥2 additional LTCs. Figures are numbers (%).

				Number of LTCs					
		Total (N=5625)		none (N=1369)		1 (N=1690)		≥2 (N=2566)	
Characteristic		n	(%)	n	(%)	n	(%)	n	(%)
Sex	Women	3928	(69.8)	968	(70.7)	1168	(69.1)	1792	(69.8)
Age group (years)	<50	669	(11.9)	254	(18.6)	214	(12.7)	201	(7.8)
	50-<60	1792	(31.9)	479	(35.0)	550	(32.5)	763	(29.7)
	60+	3164	(56.2)	636	(46.5)	926	(54.8)	1602	(62.4)
BMI (kg/m^2^)	<18.5	50	(0.9)	17	(1.2)	21	(1.2)	12	(0.5)
	18.5-<25	1586	(28.2)	497	(36.3)	501	(29.6)	588	(22.9)
	25-<30	2161	(38.4)	566	(41.3)	694	(41.1)	901	(35.1)
	30+	1775	(31.6)	282	(20.6)	460	(27.2)	1033	(40.3)
	Missing	53	(0.9)	7	(0.5)	14	(0.8)	32	(1.2)
Townsend scores	1 least deprived	995	(17.7)	294	(21.5)	329	(19.5)	372	(14.5)
	2	975	(17.3)	247	(18.0)	307	(18.2)	421	(16.4)
	3	1086	(19.3)	296	(21.6)	338	(20.0)	452	(17.6)
	4	1146	(20.4)	273	(19.9)	346	(20.5)	527	(20.5)
	5 - most deprived	1413	(25.1)	259	(18.9)	365	(21.6)	789	(30.7)
	Missing	10	(0.2)	<5	(<0.4)	5	(0.3)	5	(0.2)
Smoking history	never smoked	2618	(46.5)	720	(52.6)	805	(47.6)	1093	(42.6)
	current/previous	2960	(52.6)	641	(46.8)	871	(51.5)	1448	(56.4)
	Missing	47	(0.8)	8	(0.6)	14	(0.8)	25	(1.0)
Alcohol consumption	never/special occasions	1818	(32.3)	326	(23.8)	497	(29.4)	995	(38.8)
	1 to 3 times a month	683	(12.1)	179	(13.1)	201	(11.9)	303	(11.8)
	at least once a week	3114	(55.4)	862	(63.0)	988	(58.5)	1264	(49.3)
	Missing	10	(0.2)	<5	(<0.4)	<5	(<0.3)	<5	(<0.2)
Physical activity	none	809	(14.4)	143	(10.4)	202	(12.0)	464	(18.1)
	low	406	(7.2)	81	(5.9)	110	(6.5)	215	(8.4)
	medium	4091	(72.7)	1061	(77.5)	1279	(75.7)	1751	(68.2)
	high	182	(3.2)	72	(5.3)	61	(3.6)	49	(1.9)
	Missing	137	(2.4)	12	(0.9)	38	(2.2)	87	(3.4)

In total 1,369 (24%) participants reported no other LTC, 1,690 (30%) reported one and 2,566 (46%) reported 2 or more LTCs. Participants who reported 2 or more LTCs were older, had a higher prevalence of smoking, obesity, low physical activity, and a larger proportion were from the most deprived quintile of area deprivation scores (Table 1).

### LTCs

**Figure 1.**
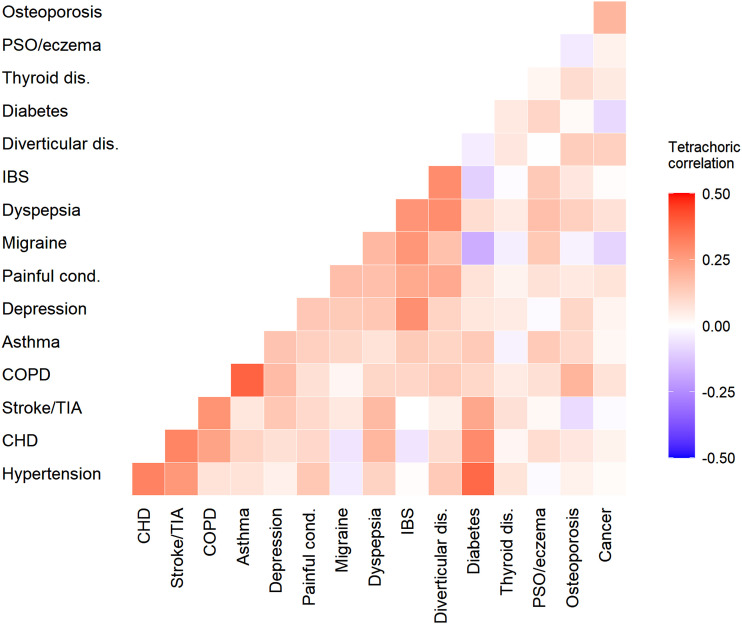
Association between additional long-term conditions (LTCs) in persons with RA. Heatmap indicating the tetrachoric correlations between LTCs. Data are shown for the 16 LTCs which had a prevalence ≥2% in the study sample. CHD-coronary heart disease, COPD-chronic obstructive pulmonary disease, Diverticular dis.-diverticular disease, IBS-irritable bowel syndrome, Painful cond.-painful conditions; PSO/eczema-psoriasis or eczema, Stroke/TIA-stroke or transient ischaemic attack, Thyroid dis.-thyroid disorders.

The prevalence of LTCs ranged from 36% and 19% for hypertension and painful conditions respectively, to less than 0.1% for each of constipation, dementia, anorexia/bulimia, and abuse of psychoactive drugs (Supplementary Table S3). Figure 1 shows the tetrachoric correlations between the 16 conditions which had a prevalence of at least 2% in the study sample. The correlations suggested a moderate tendency for some conditions to be associated. Correlations were strongest between COPD and asthma (correlation 0.38); coronary heart disease (CHD), hypertension, and stroke (correlations between 0.27-0.32); irritable bowel syndrome (IBS) with depression (0.29) and migraine (0.27); IBS, dyspepsia, and diverticular disease (0.27-0.29). Cancer and thyroid conditions did not display marked associations with other LTCs.

**Figure 2.**
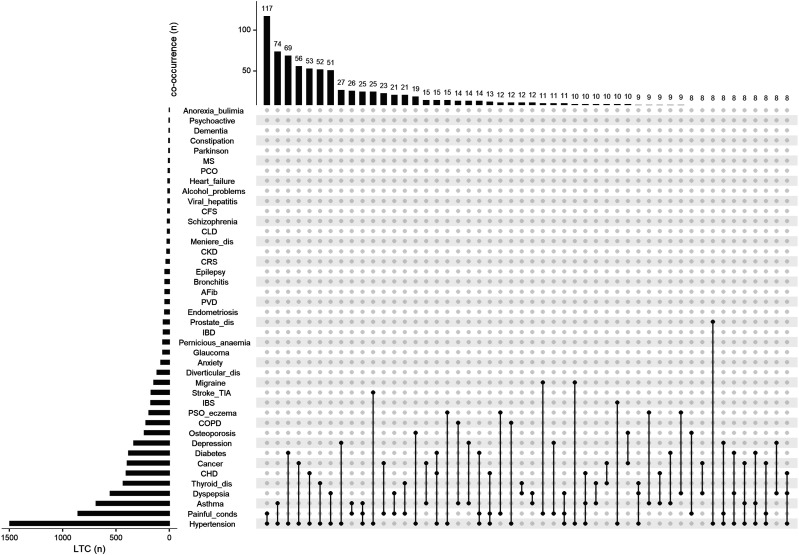
The 50 most common combinations of long-term conditions (LTCs) in persons with RA and ≥2LTCs. The 50 most common combinations of LTCs ranked by prevalence. Black dots and lines indicate specific combination of LTCs. The top bar-chart indicates the number of participants who reported each combination of LTCs. The side bar-chart indicates the number of participants who reported each LTC. AFib-arterial fibrillation, CFS-chronic fatigue syndrome, CHD-coronary heart disease, CKD-chronic kidney disease, CLD-chronic liver disease, COPD-chronic obstructive pulmonary disease, CRS-chronic sinusitis, Diverticular_dis.-diverticular disease, IBD-inflammatory bowel disease, IBS-irritable bowel syndrome, Meniere_dis.-Meniere's disease, MS-multiple sclerosis, Painful_conds-painful conditions, PCOS-polycystic ovary syndrome, PSO_eczema-psoriasis or eczema, Psychoactive-psychoactive substance abuse, PVD-peripheral vascular disease, Stroke_TIA-stroke or transient ischaemic attack, Thyroid_dis.-thyroid disorders.

Among the 1,690 participants who reported only one additional LTC, the three most common single conditions were hypertension (n=507, 30.0%), painful conditions (definition Supplementary Table S1) (210, 12.4%) and asthma (174, 10.3%) (Supplementary Table S3). Among the 2,566 participants who reported 2 or more LTCs, there were 1,138 distinct combinations of LTCs (Supplementary Figure S1). Of these distinct combinations, 86% (984) were reported by no more than 2 individuals; in total these combinations involved 1,118 (44%) participants. The remaining 154 (14%) LTC combinations were reported by 1,448 (56%) participants in total. The three most common combinations comprised RA plus hypertension with each of painful conditions (n=117, 4.6%), asthma (74, 2.9%), and diabetes (69, 2.7%). Figure 2 provides details of the 50 most common combinations.

### Latent class analysis

A five-class solution was selected from the LCA. This was a pragmatic choice because the behaviour of the BIC did not suggest an optimal number of classes (Supplementary Figure S2) whereas the BIC in the analysis of 16 LTCs indicated a possible 5 cluster solution (Supplementary Figure S3). Both analyses assigned 89% of participants to equivalent classes. Entropy was 0.64 for the 5-class solution and this did not change substantially between a 4 (0.56) or 6 (0.65) class solution. The size of the classes ranged from 11% to 38% of persons with RA and ≥2 additional LTCs. Hypertension and painful conditions were highly prevalent in all classes, and the majority of LTCs were present in each class. A summary of the characteristics of each class is shown in Table 2 (a full description is provided in Supplementary Tables S4-S5 & Supplementary Figures S4-S9). The lead distinctive condition in classes 1-5 respectively were thyroid disorders (class 1; n=354, 14% of participants with RA and ≥2 additional LTCs), painful conditions (class 2; n=428, 17%), asthma (class 3; n=514, 20%), hypertension (class 4 -the most common class; n=983, 38%) and cancer (class 5; n=287, 11%).

**Table 2.** Characteristics of participants in each latent class. For ease of reference up to three most distinctive conditions are shown for each class. All LTCs can be prevalent to varying degrees within each class, for example painful conditions and hypertension are a feature of all classes. Distinctive conditions are those which appear to distinguish classes from one another. A detailed description of participant characteristics and the prevalence of each LTC within each class and their combinations is provided in Supplementary Tables S4-S5 & Supplementary Figures S4-S9.

Class	3 most distinctive conditions (within class prevalence)		Participants n (%)	Median number of conditions in addition to RA (IQR)	Number of distinct combinations of LTCs	Women (%)	Age 60+ years (%)	BMI>30 (%)	Current or previous smoker (%)	Reside in most deprived quintile (%)
	Lead condition (%)	other conditions^a^								
1	Thyroid disorders (100.0%)		354 (13.8)	3 (2-4)	173	94.4	62.7	38.1	47.5	28.5
2	Painful conditions (55.8%)	Dyspepsia (40.2%), migraine (28.7%)	428 (16.7)	3 (2-4)	257	78	52.1	32	54	31.8
3	Asthma (60.9%)	COPD (30.4%), CHD (23.0%)	514 (20.0)	3 (2-4)	334	64	60.7	36.4	61.5	35.4
4	Hypertension (100%)	Diabetes (25%), CHD (22.4%)	983 (38.3)	2 (2-3)	266	60.4	64.8	49	58.4	30.8
5	Cancer (100%)		287 (11.2)	2 (2-3)	108	70	72.5	32.1	55.4	23.3

^a^no other clear distinctive conditions were identified in classes 1 and 5.

Participants in class 1, whose most distinctive condition was thyroid disorders (within-class prevalence 100%), were predominately women (94%) and had a median of three additional LTCs. No other distinctive conditions could be clearly identified. The two most common combinations involved thyroid disorders with hypertension (within-class prevalence 15%) or painful conditions (6%).

Painful conditions (within-class prevalence 56%), migraine (29%) and dyspepsia (40%) were the 3 most distinctive conditions in class 2. This class also displayed a high prevalence of IBS (26%), depression (24%), diverticular disease (11%) and anxiety (9%). It contained younger participants (48% aged<60 years) and 78% were women. The median number of conditions additional to RA was 3. The most common combination was painful conditions with dyspepsia (5%).

Class 3 (distinctive conditions asthma (61%), COPD (30%), and CHD (23%)) had a high prevalence of current or past smoking (62%) and 64% were women. This class also had a raised prevalence of osteoporosis (18%), diabetes (19%) and depression (16%). The most common combination in this class was asthma with painful conditions (5%).

The prominent condition in the largest class (class 4) was hypertension (100%). The other distinctive conditions in this class were diabetes (25%) and CHD (22%). This class had the highest proportion of men (40%) and participants who were obese (49%) while 58% had a history of smoking and 31% came from the most deprived quintile of deprivation scores. The most common combination was hypertension with painful conditions (12%).

All persons in the smallest class (class 5) had a life-time cancer diagnosis (within class prevalence 100%) in addition to RA. No other distinctive conditions could be clearly identified. This class contained the highest proportion of participants aged ≥60 years (73%) with a median of 2 conditions in addition to RA. The most common combination was cancer with hypertension (20%).

### Adverse Health Outcomes

Numbers of deaths, MACE and emergency hospitalisations are shown in Table 3.

**Table 3.** Adverse health outcomes among RA participants. Hazard ratios for all-cause mortality and MACE, admission rate ratios for number of emergency hospitalisations. RA participants with ≥2LTCs categorised into 5 classes using latent class analysis. All LTCs are prevalent to varying degrees within each class. The prevalence of each LTC within each class is provided in Supplementary Tables S4-S5 & Supplementary Figures S4-S9. The 3 most distinctive conditions in each class were: class 1-thyroid disorders; class 2-painful conditions, dyspepsia, migraine; class 3-asthma, COPD, CHD; class 4-hypertension, diabetes, CHD; class 5-cancer. Outcomes are expressed relative to RA participants without any other LTCs.

				Hazard ratio or admission rate ratio (95% CI)					
Outcome	LTC group	Number of events	Events per 100 person years	adjusted for sex & age		adjusted for sex, age, smoking & deprivation		adjusted for sex, age, smoking, deprivation, BMI, alcohol & physical activity	
All-cause mortality	none	92	0.62	1		1		1	
	one LTC	144	0.79	1.11	(0.85-1.44)	1.10	(0.84-1.43)	1.08	(0.82-1.41)
	class 1	43	1.13	1.62	(1.13-2.34)	1.51	(1.04-2.19)	1.53	(1.04-2.25)
	class 2	37	0.79	1.21	(0.83-1.78)	1.14	(0.78-1.67)	1.08	(0.73-1.61)
	class 3	107	2.02	2.71	(2.04-3.58)	2.44	(1.84-3.25)	2.32	(1.73-3.11)
	class 4	161	1.56	1.85	(1.43-2.40)	1.71	(1.31-2.22)	1.64	(1.25-2.16)
	class 5	63	2.22	2.57	(1.86-3.55)	2.56	(1.85-3.54)	2.66	(1.91-3.70)
	All	647	1.08						
Major adverse cardiovascular events (MACE)	none	66	0.45	1		1		1	
	one LTC	150	0.86	1.72	(1.29-2.3)	1.69	(1.26-2.26)	1.72	(1.28-2.31)
	class 1	34	0.93	2.29	(1.50-3.4)	2.16	(1.41-3.30)	2.25	(1.46-3.46)
	class 2	34	0.76	1.69	(1.11-4.5)	1.65	(1.09-2.51)	1.59	(1.04-2.43)
	class 3	85	1.73	3.27	(2.37-5.5)	3.12	(2.25-4.32)	2.95	(2.11-4.14)
	class 4	163	1.71	2.93	(2.20-6.9)	2.76	(2.06-3.70)	2.71	(2.00-3.66)
	class 5	31	1.15	2.01	(1.31-7.0)	2.02	(1.31-3.10)	2.07	(1.34-3.21)
	All	563	0.98						
Emergency hospital admission	none	1308	8.85	1		1		1	
	one LTC	2252	12.44	1.38	(1.23-1.56)	1.36	(1.21-1.54)	1.32	(1.17-1.49)
	class 1	673	17.87	2.01	(1.67-2.42)	1.98	(1.64-2.39)	1.84	(1.52-2.23)
	class 2	752	16.25	1.83	(1.54-2.17)	1.74	(1.46-2.07)	1.59	(1.33-1.90)
	class 3	1626	31.01	3.75	(3.20-4.40)	3.39	(2.89-3.98)	3.01	(2.56-3.54)
	class 4	2356	23.06	2.53	(2.22-2.89)	2.35	(2.06-2.69)	2.05	(1.79-2.36)
	class 5	632	22.39	2.90	(2.37-3.56)	2.82	(2.30-3.45)	2.75	(2.24-3.37)
	All	9599	16.12						

### All-cause mortality

A total of 647 (11%) participants with RA died over a median follow-up of 11.1 years. The crude mortality rate was 1.1 deaths per 100 person years. Table 3 shows that the highest mortality was observed in class 5 (lifetime cancer diagnosis as the distinctive condition; 22% died), and in class 3 (distinctive conditions asthma, COPD, and CHD) in which 21% died (HR 2.66 (95%CI 1.91-3.70) and 2.32 (1.73-3.11) respectively, compared to participants with RA and no other LTC and adjusted for sex, age, deprivation, BMI, alcohol consumption and physical activity). Class 2 (painful conditions, migraine, and dyspepsia; median number of 3 LTCs in addition to RA) had an adjusted overall risk of death similar to RA participants with one LTC (adjusted HR 1.08 (95%CI 0.73-1.61) and 1.08 (0.82-1.41) respectively).

### Major adverse cardiovascular events (MACE)

A total of 589 (10%) participants experienced a MACE over a median follow-up of 10.8 years. All classes showed a substantial increased risk of MACE compared to persons with RA and no other LTC (Table 3). The highest risk appeared in the two classes which had a high prevalence of CHD; class 3 (distinctive conditions asthma, COPD and CHD), and class 4 (hypertension, CHD, and diabetes). In both these classes 17% experienced a MACE during follow-up. The fully adjusted hazard ratios in these classes were 2.80 (95%CI 2.02-3.87) and 2.54 (1.90-3.41) respectively.

### Emergency hospitalisation

There was a total of 9,545 emergency hospital admissions involving 3,101 (55%) participants over a median follow-up of 11.0 years. The crude admission rate was 16 admissions per 100 person years. The highest rate was observed in class 3 (distinctive conditions asthma, COPD and CHD) in which 73% had at least one emergency admission (Table 3). The adjusted admission rate ratio for this class was 3.01 (95%CI 2.56-3.54) compared to participants with RA and no other LTC.

### Outcomes based on number of LTCs within classes

**Figure 3.**
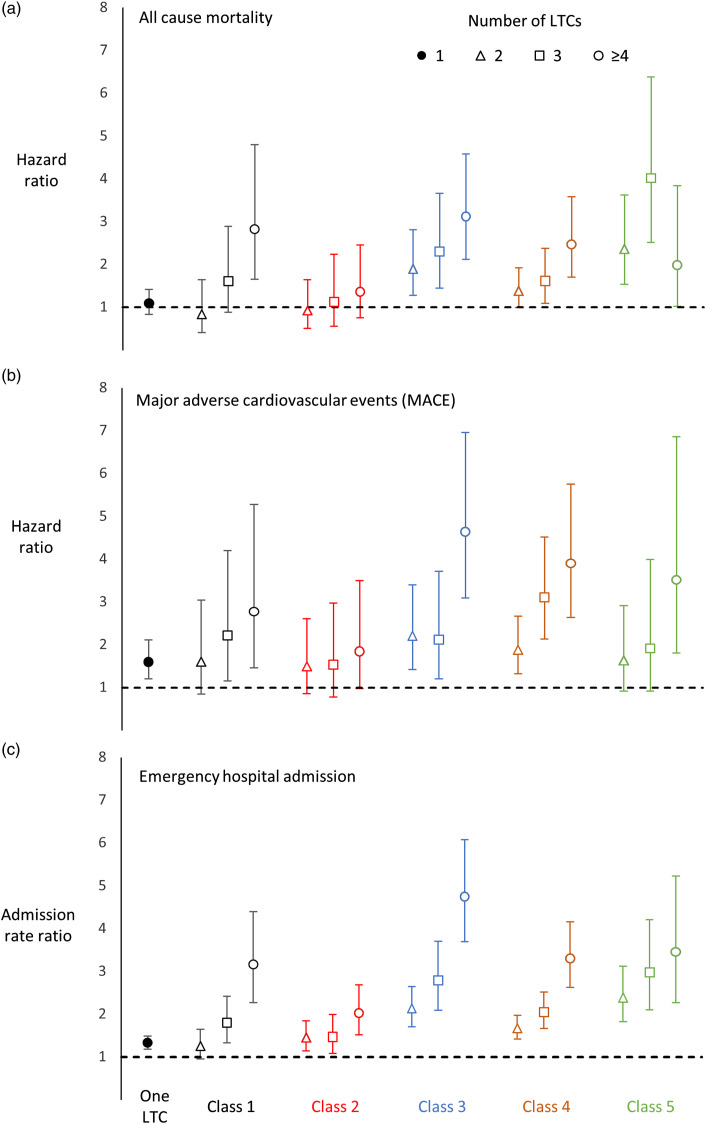
Adverse events in each class by number of long-term conditions (LTCs). Hazard ratios (95%CI) by number of LTCs (2, 3, ≥4) for (A) all-cause mortality and (B) major adverse cardiac events. Admission rate ratios (C) for number of emergency hospital admissions. Ratios adjusted for sex, age, deprivation, smoking, BMI, alcohol consumption and physical activity. Reference group is RA participants with no LTC. Prevalence of each LTC within each class is provided in Supplementary Tables S4-S5 & Supplementary Figures S4-S9. The 3 most distinctive conditions in each class were: class 1-thyroid disorders; class 2-painful conditions, dyspepsia, migraine; class 3-asthma, COPD, CHD; class 4-hypertension, diabetes, CHD; class 5-cancer.

Individuals within each latent class had varying combinations and numbers of LTCs. Figure 3 shows outcomes for each class subdivided by the number of LTCs reported by class members. There was a corresponding general rise in mortality, MACE and emergency hospital admissions within each class as the number of LTCs increased. However, the overall pattern was complex. For those who had 2 conditions in class 1 or 2 the hazard of mortality was not different to RA participants with no LTCs (HR 0.83 (95%CI 0.42-1.65) and 0.92 (0.51-1.65), respectively). Mortality in class 2 for those with 4 or more conditions (HR 1.36 (95%CI 0.75-2.45)) was as low or lower than that experienced among those with 2 conditions in classes 3, 4 and 5 (HRs 1.90 (1.28-2.81), 1.38 (0.99-1.93), 2.36 (1.54-3.63), respectively). Class 2 did not show a substantial increased risk of MACE with increasing numbers of LTCs. For those with 3 LTCs in class 2 the hazard remained slightly lower than that among those with 1 LTC (HR 1.60 (95%CI 1.20-2.12)).

When individual LTCs which had been reported by 20 or more individuals were examined (Supplementary Table S6), notable increased hazards of death compared to participants with no other LTC were found among those who had reported the following individual conditions alone - cancer (HR 2.08 (95%CI (1.22-3.56)), CHD (2.56 (1.42-4.60)), and COPD (2.17 (0.94-4.99)).

## Discussion

This large study used LCA and a broad range of LTCs to derive five classes of persons with RA and multimorbidity. Our analysis showed differences in participant characteristics across morbidity classes consistent with the literature on specific LTCs. For example, class 1 was associated with thyroid disorders and mainly involved women,^24^ class 4 had high levels of cardiometabolic conditions and contained the highest percentage of men and obesity,^25^ class 5 was the oldest and all had had a cancer diagnosis.^26^ The risk of mortality in class 5 was over 2.5 times that observed among RA participants with no other LTC. The risk of MACE and emergency hospitalisations in class 3 (which comprised asthma, COPD & CHD) was three times that experienced among participants with RA and no other LTC.

Our findings resonate with literature on RA and increased adverse health outcomes risk associated with the presence of additional LTCs. Examples include the importance of cardiovascular disease in RA both as a single condition or in combination with other conditions.^27^ Other inflammatory conditions, such as COPD, asthma, and thyroid disorders, have previously been found to have an increased prevalence in persons with RA and be associated with adverse outcomes.^28,29^ In our study, we identified two classes in which CHD was a distinctive condition – in one class (class 3) with obstructive lung disease, and in another (class 4) with CHD risk factors, diabetes and hypertension. This observation agrees with England et al^30^ who identified cardiometabolic, cardiopulmonary, and mental health/pain disorders as the predominant dimensions of multimorbidity in RA, which are reflected in classes 2-4, in our data. Our study expands this work by assigning persons with RA to morbidity classes and demonstrating the association with adverse outcomes in each class. It was apparent that the risk of adverse health outcomes was higher in classes with cancer, cardiovascular and respiratory LTCs. In addition, within each class, the risk generally increased with the number of conditions, which is in line with other studies.^18^

Interestingly, class 2 exhibited a higher prevalence of IBS, migraine, depression, anxiety, and painful conditions, all of which have individually been shown to be associated with each other, with fibromyalgia and with RA.^31,32^ Our research extends this by demonstrating their combined impact. While class 2 had a median of 3 LTCs, the impact on adverse outcomes was similar to that found among those with RA plus 1 LTC. This class displayed a weaker gradient in mortality, emergency hospitalisation and MACE as number of LTCs increased. This has not been studied before. It is an interesting finding because over a quarter of persons in class 2 had depression which has been shown to be associated with MACE.^33^

### Strengths and limitations

A limitation of our study is that the UK Biobank cohort is not a representative population sample. The decision to participate may have been influenced by an individual’s health experience and the type or severity of any LTCs they may have. LTCs were self-reported, and we had no indication of severity or duration. Nevertheless, risk factor associations in UK Biobank appear to be generalisable.^34^ The sample size, while large, was too small to provide informative estimates of outcomes for most distinct LTC combinations. Most combinations were reported by 1 or 2 individuals, and only a small number of combinations were reported by a sizeable group.

LCA is a form of cluster analysis. Cluster analysis is an exploratory technique, and different methods may produce different results when associations between conditions are weak, or when the conditions or prevalence vary.^35^ As more health conditions are compared, individuals become more dissimilar; as the prevalence of conditions decreases, the number of individuals experiencing specific combinations becomes sparse. The classes we have presented were obtained through a systematic process using LCA. Zhu et al^36^ used LCA to derive 20 multimorbidity clusters across four age strata in general practice patients in England, while Wu et al^37^ employed it to derive four comorbidity classes for patients with psoriasis in Taiwan. Previous analyses of RA have been limited to the classification of clinical features of the disease and the identification of phenotypes.^38–40^ Our analysis is somewhat different in that we considered a broad range of LTCs which are indicative of health care need in the general population. As far as we know, ours is the first study to use LCA to categorise persons with RA using such a broad range of additional LTCs. A major difficulty, however, is interpreting or understanding what derived classes may represent, especially as there is substantial heterogeneity in their composition. There is also a large degree of uncertainty in the assignment of individuals to a particular class. Clustering methods can be useful heuristic tools for revealing underlying patterns but if it is not easily understood what derived classes really represent then their clinical utility is limited.

The strength of this study is that it involved a large nationwide cohort, with high-quality data recording and a long follow-up for hospitalisation and mortality. We employed a wide range of conditions, chosen for their burden in the general population rather than association with RA. We did not merely rely on counts of LTCs but explored the associations between conditions and the distribution of their combination. This approach to multimorbidity analysis has been described as centred on the patient, rather than on specific diseases.^15,30^

In conclusion, we observed five classes of participants with ≥2 additional LTCs in RA. The risk of mortality and cardiovascular events were consistently higher in four classes compared to those with RA alone or with 1 additional LTC. Morbidity classes distinguished by cancer, cardiovascular, and respiratory LTCs were at significantly higher risk of adverse outcomes. Importantly, within most classes, the risk of adverse outcomes was higher with an increase in the number of LTCs. These findings help unpack the complex relationship between type and number of LTCs with the risk of adverse outcomes among persons with RA. Patterns of multimorbidity should be considered in risk assessment and formulating management plans in patients with RA.

## Supplemental Material

Supplemental Material - Classification of long-term condition patterns in rheumatoid arthritis and associations with adverse health events: a UK Biobank cohort study Click here for additional data file. Supplemental Material for Classification of long-term condition patterns in rheumatoid arthritis and associations with adverse health events: a UK Biobank cohort study by Philip McLoone, BSc, Bhautesh D Jani, MD, PhD, Stefan Siebert, MD, PhD, Fraser R Morton, MRes, Jordan Canning, MRes, Sara Macdonald, PhD, Frances S Mair, MD, DRCOG, FRCGP and Barbara I Nichol, PhD in Journal of Multimorbidity and Comorbidity
