# Supplementary material for: Classification of long-term condition patterns in rheumatoid arthritis and associations with adverse health events: a UK Biobank cohort study
Source: J Multimorb Comorb. 2023 Feb 10;13:26335565221148616. doi: 10.1177/26335565221148616 (PMC9926377; doi:10.1177/26335565221148616)
Supplement: Supplemental Material - Classification of long-term condition patterns in rheumatoid arthritis and associations with adverse health events: a UK Biobank cohort study [file sj-pdf-1-cob-10.1177_26335565221148616.pdf]

Classification of multimorbidity patterns in rheumatoid arthritis and associations with adverse health events: a UK Biobank study

The following tables and figures provide more details of the analysis performed in this study.

Supplementary Tables

Table S1 - Details of 42 long-term conditions (LTCs) used in the latent class analysis (LCA).

Table S2 - Details of 16 long-term conditions (LTCs) used in the secondary latent class analysis (LCA).

Table S3 - Prevalence of long-term conditions (LTCs) amongst those with RA, prevalence among those with 1 additional LTC and among those with ≥2 additional LTCs.

Table S4 - Prevalence of each long-term condition (LTC) within each latent class.

Table S5 - Characteristics of participants assigned to each of the 5 latent classes.

Table S6 - Hazard ratios for all-cause mortality for LTC combinations reported by ≥20 participants.

Supplementary Figures

Figure S1 - Illustration of all long-term conditions (LTCs) combinations in persons with RA and ≥2 LTCs.

Figure S2 - Statistical indices (sample size-adjusted Bayesian Information Criteria (BIC), entropy for classification quality, and likelihood ratio) by number of latent classes using 42 LTCs.

Figure S3 - Statistical indices (sample size-adjusted Bayesian Information Criteria (BIC), entropy for classification quality, and likelihood ratio) by number of latent classes using 16 LTCs.

Figure S4 - Prevalence of each long-term condition (LTC) within each latent class.

Figures S5 to S9 - Illustration of the 50 most prevalent long-term condition (LTC) combinations in each latent class.

**Long term condition grouping**	**Conditions included as reported by participants**
1. Alcohol problems	Alcohol dependency<br>Alcoholic liver disease/alcoholic cirrhosis
2. Anxiety	Anxiety/panic attacks<br>Nervous breakdown<br>Post-traumatic stress disorder<br>Obsessive compulsive disorder<br>Stress<br>Insomnia<br>Psychological/psychiatric problem
3. Anorexia or bulimia	Anorexia<br>Bulimia<br>Other eating disorders
4. Asthma	Asthma
5. Atrial Fibrillation	Atrial Fibrillation
6. Bronchiectasis	Bronchiectasis
7. Cancer	Lifetime diagnosis
8. Coronary Heart Disease	Heart attack/Myocardial Infarction<br>Angina
9. Chronic fatigue syndrome	Chronic fatigue syndrome
10. Chronic kidney disease	Polycystic kidney<br>Diabetic nephropathy<br>Renal/kidney failure<br>Renal failure requiring dialysis<br>Renal failure not requiring dialysis<br>Kidney nephropathy<br>Immunoglobulin A (IgA) nephropathy
11. Chronic Liver disease	Oesophageal varices<br>Non infective hepatitis<br>Liver failure/cirrhosis<br>Primary biliary cirrhosis
12. Chronic Obstructive Pulmonary Disease (COPD)	COPD/chronic obstructive airways disease<br>Emphysema/chronic bronchitis<br>Emphysema
13. Chronic sinusitis	Chronic sinusitis

Table S1 - Details of 42 long-term conditions (LTCs) used in the latent class analysis (LCA) (continued on next page)

**Long term condition grouping**	**Conditions included as reported by participants**
14. Dementia	Dementia<br>Alzheimer’s disease<br>Cognitive impairment
15. Depression	Depression<br>Postnatal Depression
16. Diabetes	Diabetic nephropathy<br>Diabetic neuropathy/ulcers<br>Diabetes<br>Type 1 diabetes<br>Type 2 diabetes<br>Diabetic eye disease
17. Diverticular disease	Diverticular disease<br>Diverticulitis
18. Dyspepsia	Gastro-oesophageal reflux (GORD)/gastric reflux<br>Oesophagitis /Barrett's oesophagus<br>Gastric stomach ulcers<br>Gastric erosions/gastritis<br>Duodenal ulcer<br>Dyspepsia/indigestion<br>Hiatus hernia<br>Helicobacter pylori
19. Endometriosis	Endometriosis
20. Epilepsy	Epilepsy
21. Glaucoma	Glaucoma
22. Heart failure	Cardiomyopathy<br>Hypertrophic cardiomyopathy<br>Heart failure/pulmonary oedema
23. Hypertension	Hypertension<br>Essential Hypertension
24. Irritable bowel syndrome	Irritable bowel syndrome
25. Inflammatory Bowel Disease	Inflammatory Bowel Disease<br>Crohn’s disease<br>Ulcerative colitis

Table S1 - Details of 42 LTCs used in the LCA (cont.)

Long term condition grouping	Conditions included as reported by participants
26. Meniere's disease	Meniere's disease
27. Migraine	Migraine
28. Multiple Sclerosis	Multiple Sclerosis
29. Osteoporosis	Osteoporosis
30. Other psychoactive substance abuse	Opioid dependency<br>Other substance abuse/dependency
31. Painful conditions	Back pain<br>Joint pain<br>Back pain<br>Joint pain<br>Headaches (not migraine)<br>Sciatica<br>Plantar fasciitis<br>Carpal tunnel syndrome<br>Fibromyalgia<br>Arthritis<br>Shingles<br>Disc problem<br>Prolapsed disc/slipped disc<br>Spine arthritis/spondylitis<br>Ankylosing spondylitis<br>Back problem<br>Osteoarthritis<br>Gout<br>Cervical spondylosis<br>Trigeminal neuralgia<br>Disc degeneration<br>Trapped nerve/compressed nerve
32. Parkinson's disease	Parkinson's disease
33. Peripheral vascular disease	Peripheral vascular disease<br>Leg claudication/intermittent claudication
34. Pernicious Anaemia	Pernicious Anaemia
35. Polycystic ovary syndrome	Polycystic ovary

Table S1 - Details of 42 LTCs used in the LCA (cont.)

Long term condition grouping	Conditions included as reported by participants
36. Prostate disorders	Prostate problem (not cancer)<br>Enlarged prostate<br>Benign prostatic hypertrophy
37. Psoriasis/eczema	Eczema<br>Dermatitis<br>Psoriasis
38. Stroke/Transient Ischaemic Attack (TIA)	Stroke<br>TIA<br>Subarachnoid haemorrhage<br>Brain haemorrhage<br>Ischaemic stroke
39. Schizophrenia/bipolar disorder	Schizophrenia<br>Mania/<br>Bipolar disorder<br>Manic depression
40. Thyroid disorders	Thyroid problem (not cancer)<br>Hyperthyroidism/thyrotoxicosis<br>Hypothyroidism/myxoedema<br>Grave’s disease<br>Thyroid goitre<br>Thyroiditis
41. Treated constipation	Constipation
42. Viral Hepatitis	Infective/viral hepatitis<br>Hepatitis B<br>Hepatitis C<br>Hepatitis D<br>Hepatitis E

Table S1 - Details of 42 LTCs used in the LCA (cont.)

Table S2 The 16 long-term conditions (LTCs) with a prevalence of 2% or greater used in the secondary latent class analysis (LCA)

Condition
Asthma
Cancer
Chronic obstructive pulmonary disease (COPD)
Coronary heart disease (CHD)
Depression
Diabetes
Diverticular disease
Dyspepsia
Hypertension
Irritable bowel syndrome (IBS)
Migraine
Osteoporosis
Painful conditions
Psoriasis/eczema (PSO/
Stroke/Transient ischaemic attack
Thyroid disorder

		Number of LTCs	
	Total (N=5625)	One (N=1690)	Two or more (N=2566)
Hypertension	2003 (35.6%)	507 (30.0%)	1496 (58.3%)
Painful conditions	1067 (19.0%)	210 (12.4%)	857 (33.4%)
Asthma	860 (15.3%)	174 (10.3%)	686 (26.7%)
Dyspepsia	634 (11.3%)	80 (4.7%)	554 (21.6%)
Thyroid disorders	557 (9.9%)	124 (7.3%)	433 (16.9%)
Cancer	495 (8.8%)	102 (6.0%)	393 (15.3%)
Coronary heart disease	460 (8.2%)	57 (3.4%)	403 (15.7%)
Diabetes	427 (7.6%)	47 (2.8%)	380 (14.8%)
Depression	391 (7.0%)	59 (3.5%)	332 (12.9%)
Osteoporosis	273 (4.9%)	38 (2.2%)	235 (9.2%)
COPD	243 (4.3%)	24 (1.4%)	219 (8.5%)
Psoriasis/eczema	228 (4.1%)	37 (2.2%)	191 (7.4%)
Stroke/TIA	180 (3.2%)	11 (0.7%)	169 (6.6%)
Irritable bowel syndrome	188 (3.3%)	16 (0.9%)	172 (6.7%)
Migraine	187 (3.3%)	43 (2.5%)	144 (5.6%)
Diverticular disease	126 (2.2%)	12 (0.7%)	114 (4.4%)
Anxiety	92 (1.6%)	12 (0.7%)	80 (3.1%)
Inflammatory bowel disease	79 (1.4%)	23 (1.4%)	56 (2.2%)
Prostate disease	72 (1.3%)	17 (1.0%)	55 (2.1%)
Glaucoma	69 (1.2%)	7 (0.4%)	62 (2.4%)
Pernicious anaemia	69 (1.2%)	9 (0.5%)	60 (2.3%)
Endometriosis	52 (0.9%)	9 (0.5%)	43 (1.7%)
Epilepsy	52 (0.9%)	12 (0.7%)	40 (1.6%)
Atrial fibrillation	50 (0.9%)	8 (0.5%)	42 (1.6%)
Bronchitis	47 (0.8%)	6 (0.4%)	41 (1.6%)
Peripheral vascular disease	45 (0.8%)	3 (0.2%)	42 (1.6%)
Chronic sinusitis	41 (0.7%)	10 (0.6%)	31 (1.2%)
Meniere's disease	27 (0.5%)	6 (0.4%)	21 (0.8%)
Chronic kidney failure	24 (0.4%)	2 (0.1%)	22 (0.9%)
Chronic liver failure	23 (0.4%)	4 (0.2%)	19 (0.7%)
Schizophrenia	20 (0.4%)	3 (0.2%)	17 (0.7%)
Chronic fatigue syndrome	19 (0.3%)	3 (0.2%)	16 (0.6%)
Alcohol problems	17 (0.3%)	3 (0.2%)	14 (0.5%)
Viral hepatitis	16 (0.3%)	2 (0.1%)	14 (0.5%)
Heart failure	13 (0.2%)	0 (0.0%)	13 (0.5%)
Polycystic ovary disease	12 (0.2%)	2 (0.1%)	10 (0.4%)
Multiple sclerosis	10 (0.2%)	2 (0.1%)	8 (0.3%)
Parkinson's disease	8 (0.1%)	3 (0.2%)	5 (0.2%)
Constipation	5 (0.1%)	1 (0.1%)	4 (0.2%)
Dementia	3 (0.1%)	0 (0.0%)	3 (0.1%)
Anorexia/bulimia	3 (0.1%)	2 (0.1%)	1 (0.0%)
Psychoactive substance abuse	2 (0.0%)	0 (0.0%)	2 (0.1%)

Table S3 Prevalence of long-term conditions (LTCs) in the sample. Figures are numbers (%)

COPD-chronic obstructive pulmonary disease, Stroke/TIA- stroke or transient ischaemic attack

Table S4 Latent class analysis of 42 long-term conditions (LTCs). Within class prevalence of each LTC in the 5-class solution showing. Figures are numbers (%).

	Latent class				
	1 (N=354)	2 (N=428)	3 (N=514)	4 (N=983)	5 (N=287)
Hypertension	157 (44.4%)	117 (27.3%)	98 (19.1%)	983 (100.0%)	141 (49.1%)
Painful conditions	80 (22.6%)	239 (55.8%)	132 (25.7%)	326 (33.2%)	80 (27.9%)
Asthma	53 (15.0%)	65 (15.2%)	313 (60.9%)	208 (21.2%)	47 (16.4%)
Dyspepsia	59 (16.7%)	172 (40.2%)	109 (21.2%)	162 (16.5%)	52 (18.1%)
Thyroid disorders	354 (100.0%)	10 (2.3%)	7 (1.4%)	62 (6.3%)	0 (0.0%)
Cancer	58 (16.4%)	15 (3.5%)	29 (5.6%)	4 (0.4%)	287 (100.0%)
Coronary heart disease	24 (6.8%)	15 (3.5%)	118 (23.0%)	220 (22.4%)	26 (9.1%)
Diabetes	22 (6.2%)	5 (1.2%)	98 (19.1%)	243 (24.7%)	12 (4.2%)
Depression	38 (10.7%)	103 (24.1%)	85 (16.5%)	84 (8.5%)	22 (7.7%)
Osteoporosis	35 (9.9%)	30 (7.0%)	93 (18.1%)	52 (5.3%)	25 (8.7%)
COPD	19 (5.4%)	3 (0.7%)	156 (30.4%)	37 (3.8%)	4 (1.4%)
Psoriasis/eczema	20 (5.6%)	59 (13.8%)	55 (10.7%)	42 (4.3%)	15 (5.2%)
Stroke/TIA	6 (1.7%)	15 (3.5%)	48 (9.3%)	95 (9.7%)	5 (1.7%)
Irritable bowel syndrome	10 (2.8%)	111 (25.9%)	38 (7.4%)	2 (0.2%)	11 (3.8%)
Migraine	10 (2.8%)	123 (28.7%)	10 (1.9%)	1 (0.1%)	0 (0.0%)
Diverticular disease	13 (3.7%)	49 (11.4%)	17 (3.3%)	25 (2.5%)	10 (3.5%)
Anxiety	15 (4.2%)	38 (8.9%)	22 (4.3%)	1 (0.1%)	4 (1.4%)
inflammatory bowel disease	7 (2.0%)	16 (3.7%)	13 (2.5%)	15 (1.5%)	5 (1.7%)
Prostate disease	0 (0.0%)	11 (2.6%)	13 (2.5%)	30 (3.1%)	1 (0.3%)
Glaucoma	7 (2.0%)	10 (2.3%)	29 (5.6%)	13 (1.3%)	3 (1.0%)
Pernicious anaemia	20 (5.6%)	4 (0.9%)	19 (3.7%)	16 (1.6%)	1 (0.3%)
Endometriosis	9 (2.5%)	16 (3.7%)	1 (0.2%)	14 (1.4%)	3 (1.0%)
Epilepsy	5 (1.4%)	11 (2.6%)	16 (3.1%)	8 (0.8%)	0 (0.0%)
Atrial fibrillation	6 (1.7%)	4 (0.9%)	14 (2.7%)	16 (1.6%)	2 (0.7%)
Bronchitis	3 (0.8%)	0 (0.0%)	18 (3.5%)	13 (1.3%)	7 (2.4%)
Peripheral vascular disease	6 (1.7%)	15 (3.5%)	1 (0.2%)	16 (1.6%)	4 (1.4%)
Chronic sinusitis	0 (0.0%)	0 (0.0%)	22 (4.3%)	8 (0.8%)	1 (0.3%)
Meniere's disease	6 (1.7%)	5 (1.2%)	5 (1.0%)	4 (0.4%)	1 (0.3%)
Chronic kidney failure	2 (0.6%)	1 (0.2%)	10 (1.9%)	3 (0.3%)	3 (1.0%)
Chronic liver failure	6 (1.7%)	6 (1.4%)	4 (0.8%)	2 (0.2%)	4 (1.4%)
Schizophrenia	5 (1.4%)	5 (1.2%)	7 (1.4%)	0 (0.0%)	0 (0.0%)
Chronic fatigue syndrome	3 (0.8%)	11 (2.6%)	1 (0.2%)	0 (0.0%)	1 (0.3%)
Alcohol problems	1 (0.3%)	0 (0.0%)	13 (2.5%)	0 (0.0%)	0 (0.0%)
Viral hepatitis	2 (0.6%)	4 (0.9%)	6 (1.2%)	0 (0.0%)	2 (0.7%)
Heart failure	0 (0.0%)	0 (0.0%)	8 (1.6%)	5 (0.5%)	0 (0.0%)
Polycystic ovary disease	1 (0.3%)	8 (1.9%)	0 (0.0%)	1 (0.1%)	0 (0.0%)
Multiple sclerosis	0 (0.0%)	0 (0.0%)	3 (0.6%)	4 (0.4%)	1 (0.3%)
Parkinson's disease	0 (0.0%)	0 (0.0%)	2 (0.4%)	3 (0.3%)	0 (0.0%)
Constipation	0 (0.0%)	3 (0.7%)	0 (0.0%)	1 (0.1%)	0 (0.0%)
Dementia	0 (0.0%)	0 (0.0%)	3 (0.6%)	0 (0.0%)	0 (0.0%)
Anorexia/bulimia	1 (0.3%)	0 (0.0%)	0 (0.0%)	0 (0.0%)	0 (0.0%)
Psychoactive substance abuse	0 (0.0%)	0 (0.0%)	2 (0.4%)	0 (0.0%)	0 (0.0%)

COPD-chronic obstructive pulmonary disease, Stroke/TIA- stroke or transient ischaemic attack

		Class									
		1 (N=354)		2 (N=428)		3 (N=514)		4 (N=983)		5 (N=287)	
Number of combinations of LTCs		173		257		334		266		108	
Median (IQR) number of LTCs		3	(2-4)	3	(2-4)	3	(2-4)	2	(2-3)	2	(2-3)
Sex	women	334	(94.4)	334	(78.0)	329	(64.0)	594	(60.4)	201	(70.0)
Age group (years)	<50	32	(9.0)	45	(10.5)	60	(11.7)	55	(5.6)	9	(3.1)
	50-<60	100	(28.2)	160	(37.4)	142	(27.6)	291	(29.6)	70	(24.4)
	60+	222	(62.7)	223	(52.1)	312	(60.7)	637	(64.8)	208	(72.5)
BMI (kg/m2)	<18.5	0	(0.0)	4	(0.9)	3	(0.6)	3	(0.3)	2	(0.7)
	18.5-<25	83	(23.4)	123	(28.7)	138	(26.8)	164	(16.7)	80	(27.9)
	25-<30	128	(36.2)	160	(37.4)	182	(35.4)	322	(32.8)	109	(38.0)
	30+	135	(38.1)	137	(32.0)	187	(36.4)	482	(49.0)	92	(32.1)
	missing	8	(2.3)	4	(0.9)	4	(0.8)	12	(1.2)	4	(1.4)
Townsend score quintiles	1 - least deprived	67	(18.9)	65	(15.2)	57	(11.1)	138	(14.0)	45	(15.7)
	2	52	(14.7)	71	(16.6)	87	(16.9)	154	(15.7)	57	(19.9)
	3	53	(15.0)	77	(18.0)	91	(17.7)	177	(18.0)	54	(18.8)
	4	81	(22.9)	79	(18.5)	97	(18.9)	207	(21.1)	63	(22.0)
	5 - most deprived	101	(28.5)	136	(31.8)	182	(35.4)	303	(30.8)	67	(23.3)
	missing	0	(0.0)	0	(0.0)	0	(0.0)	4	(0.4)	1	(0.3)
Smoking history	Current/Previous	168	(47.5)	231	(54.0)	316	(61.5)	574	(58.4)	159	(55.4)
	Never	182	(51.4)	193	(45.1)	192	(37.4)	400	(40.7)	126	(43.9)
	missing	4	(1.1)	4	(0.9)	6	(1.2)	9	(0.9)	2	(0.7)
Alcohol consumption	Never/special occasions	127	(35.9)	187	(43.7)	203	(39.5)	379	(38.6)	99	(34.5)
	1 to 3 times a month	44	(12.4)	56	(13.1)	66	(12.8)	103	(10.5)	34	(11.8)
	at least once a week	182	(51.4)	185	(43.2)	243	(47.3)	500	(50.9)	154	(53.7)
	missing	1	(0.3)	0	(0.0)	2	(0.4)	1	(0.1)	0	(0.0)
Physical activity	none	63	(17.8)	70	(16.4)	113	(22.0)	181	(18.4)	37	(12.9)
	low	37	(10.5)	44	(10.3)	33	(6.4)	76	(7.7)	25	(8.7)
	medium	239	(67.5)	293	(68.5)	337	(65.6)	674	(68.6)	208	(72.5)
	high	6	(1.7)	12	(2.8)	10	(1.9)	14	(1.4)	7	(2.4)
	missing	9	(2.5)	9	(2.1)	21	(4.1)	38	(3.9)	10	(3.5)

Table S5 Characteristics of participants in each of the 5 latent classes. Figures are numbers (percentages) unless indicated otherwise

			Adjusted for age & sex		Adjusted for age, sex, BMI, deprivation, smoking, alcohol, physical activity	
Class	Conditions	N	HR	95%CI	HR	95%CI
	Hypertension only	507	1.01	(0.70-1.44)	1.06	(0.73-1.54)
	Painful conditions only	210	0.59	(0.31-1.14)	0.64	(0.33-1.24)
	Asthma only	174	0.40	(0.16-0.99)	0.43	(0.17-1.05)
	Thyroid disorders only	124	0.91	(0.42-1.97)	0.85	(0.37-1.95)
4	Hypertension & painful cond.	117	1.12	(0.61-2.05)	0.94	(0.47-1.88)
	Cancer only	102	2.14	(1.26-3.64)	2.08	(1.22-3.56)
	Dyspepsia only	80	0.85	(0.34-2.08)	0.78	(0.32-1.92)
4	Hypertension & asthma	74	1.64	(0.85-3.15)	1.86	(0.96-3.60)
4	Hypertension & diabetes	69	1.78	(0.78-4.06)	1.94	(1.03-3.66)
	Depression only	59	0.00		0.00	
	Coronary heart disease only	57	2.95	(1.70-5.10)	2.56	(1.42-4.60)
1	Thyroid disorder & hypertension	52	1.22	(0.50-3.01)	0.75	(0.24-2.39)
5	Cancer & hypertension	56	1.80	(0.91-3.58)	2.11	(1.06-4.21)
4	Hypertension & coronary herat disease	53	0.95	(0.42-2.18)	0.91	(0.40-2.10)
4	Hypertension & dyspepsia	51	1.55	(0.72-3.36)	1.46	(0.67-3.17)
	Diabetes only	47	1.78	(0.78-4.06)	2.02	(0.88-4.63)
	Migraine only	43	1.87	(0.69-5.09)	2.09	(0.77-5.71)
	Osteoporosis only	28	2.09	(0.91-4.80)	1.19	(0.43-3.28)
4	Hypertension & depression	27	1.46	(0.46-4.62)	0.96	(0.24-3.91)
	Psoriasis/eczema only	27	1.88	(0.69-5.12)	1.48	(0.47-4.69)
3	Asthma & painful conditions	26	1.54	(0.49-4.87)	1.62	(0.51-5.14)
4	Hypertension & painful conditions & asthma	25	0.45	(0.06-3.27)	0.47	(0.06-3.36)
4	Hypertension & stroke/transient ischaemic attack	25	3.78	(1.75-8.15)	3.53	(1.63-7.66)
	Chronic obstructive bowel disease only	24	3.18	(1.47-6.86)	2.17	(0.94-4.99)
	Inflammatory bowel disease only	23	0.56	(0.08-4.02)	0.64	(0.09-4.57)
5	Cancer & painful conditions	23	1.44	(0.46-4.55)	1.60	(0.50-5.06)
2	Painful conditions & dyspepsia	21	0.61	(0.08-4.37)	0.53	(0.07-3.85)
1	Thyroid disorder & painful conditions	21	1.12	(0.61-2.05)	0.79	(0.11-5.68)
	one LTC (excluding those above)	165	1.10	(0.63-1.93)	1.08	(0.60-1.93)
	two LTCs (excluding those above)	628	1.57	(1.16-2.13)	1.53	(1.12-2.08)
	three LTCs (excluding those above)	675	2.10	(1.59-2.76)	1.97	(1.47-2.63)
	four+ LTCs	623	2.85	(2.19-3.71)	2.43	(1.82-3.23)

Table S6 Hazard ratios (95%CI) for all-cause mortality for selected long-term condition (LTC) combinations reported by 20 or more participants. Reference category participants RA and no other LTCs

Figure S1 - Illustration of all long-term conditions (LTCs) combinations in persons with RA and ≥2 LTCs. Each of the 1138 distinct combinations of LTCs ranked by prevalence on the horizontal axis (high to low). Horizontal axis demarcated at combinations 1-35, 36-154, 155-1138. Total number of combinations and persons in each section are shown with the number (or range) of persons within each combination. Vertical axis ranks LTCs by prevalence. Ticks within graph indicate LTCs contained in each combination.

AFib-arterial fibrillation, CFS-chronic fatigue syndrome, CHD-coronary heart disease, CKD-chronic kidney disease, CLD-chronic liver disease, COPD-chronic obstructive pulmonary disease, CRS-chronic sinusitis, Diverticular_dis.-diverticular disease, IBD-inflammatory bowel disease, IBS-irritable bowel syndrome, Meniere_dis.-Meniere's disease, MS-multiple sclerosis, Painful_conds-painful conditions, PCOS-polycystic ovary syndrome, PSO_eczema-psoriasis or eczema, Psychoactive-psychoactive substance abuse, PVD-peripheral vascular disease, Stroke_TIA-stroke or transient ischaemic attack, Thyroid_dis.-thyroid disorders. CFS-chronic fatigue syndrome.

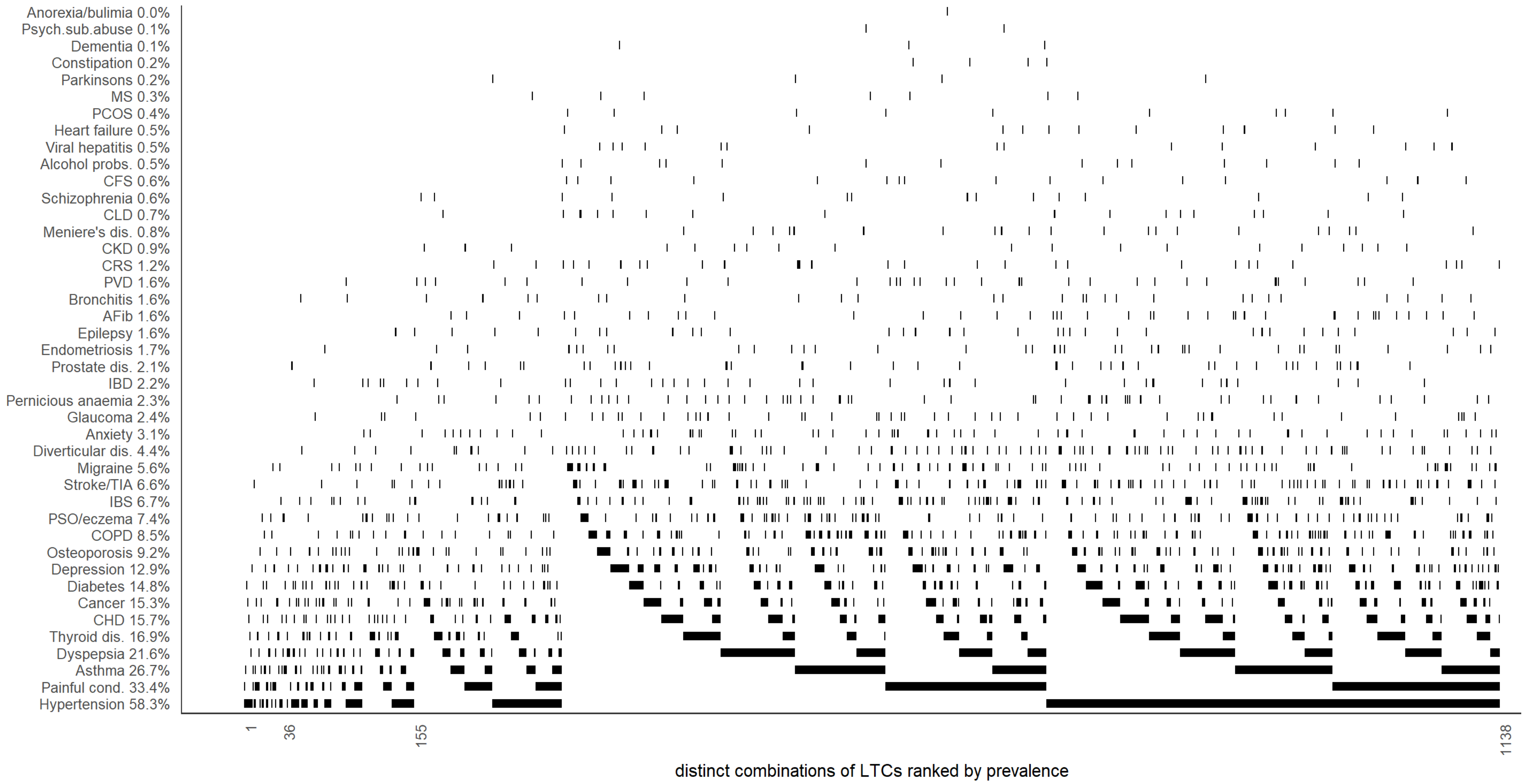

N in each combination	117-10	9-3	2-1
N (%) of combinations	35 (3%)	119 (10%)	984 (86%)
N (%) of subjects	900 (35%)	548 (21%)	1118 (44%)

Figure S1 Illustration of all long-term conditions (LTCs) combinations in persons with RA and ≥2 LTCs.

Figure S2 Statistical indices (sample size-adjusted Bayesian Information Criteria (BIC), entropy for classification quality, and likelihood ratio) by number of latent classes using 42 LTCs.

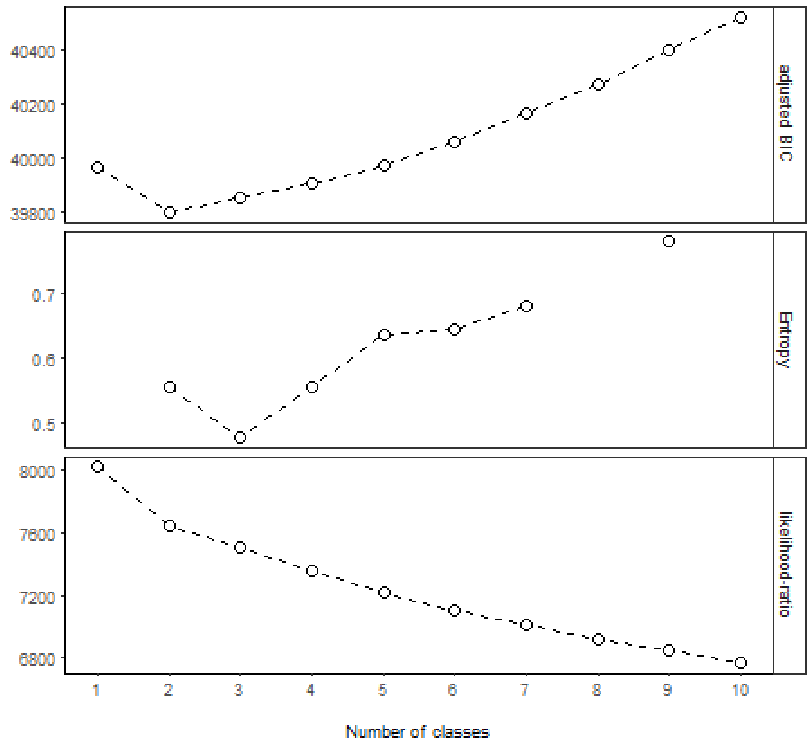

Figure S3 Behaviour of statistical indices (sample size-adjusted Bayesian Information Criteria (BIC), entropy for classification quality, and likelihood ratio) by number of latent classes using 16 LTCs with a prevalence of 2% in the UK Biobank RA sample.

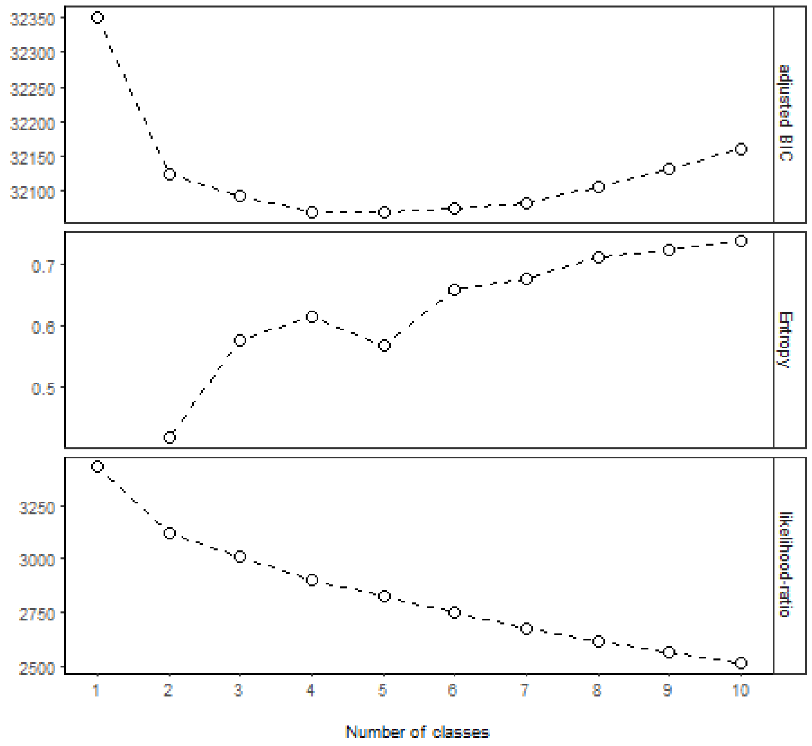

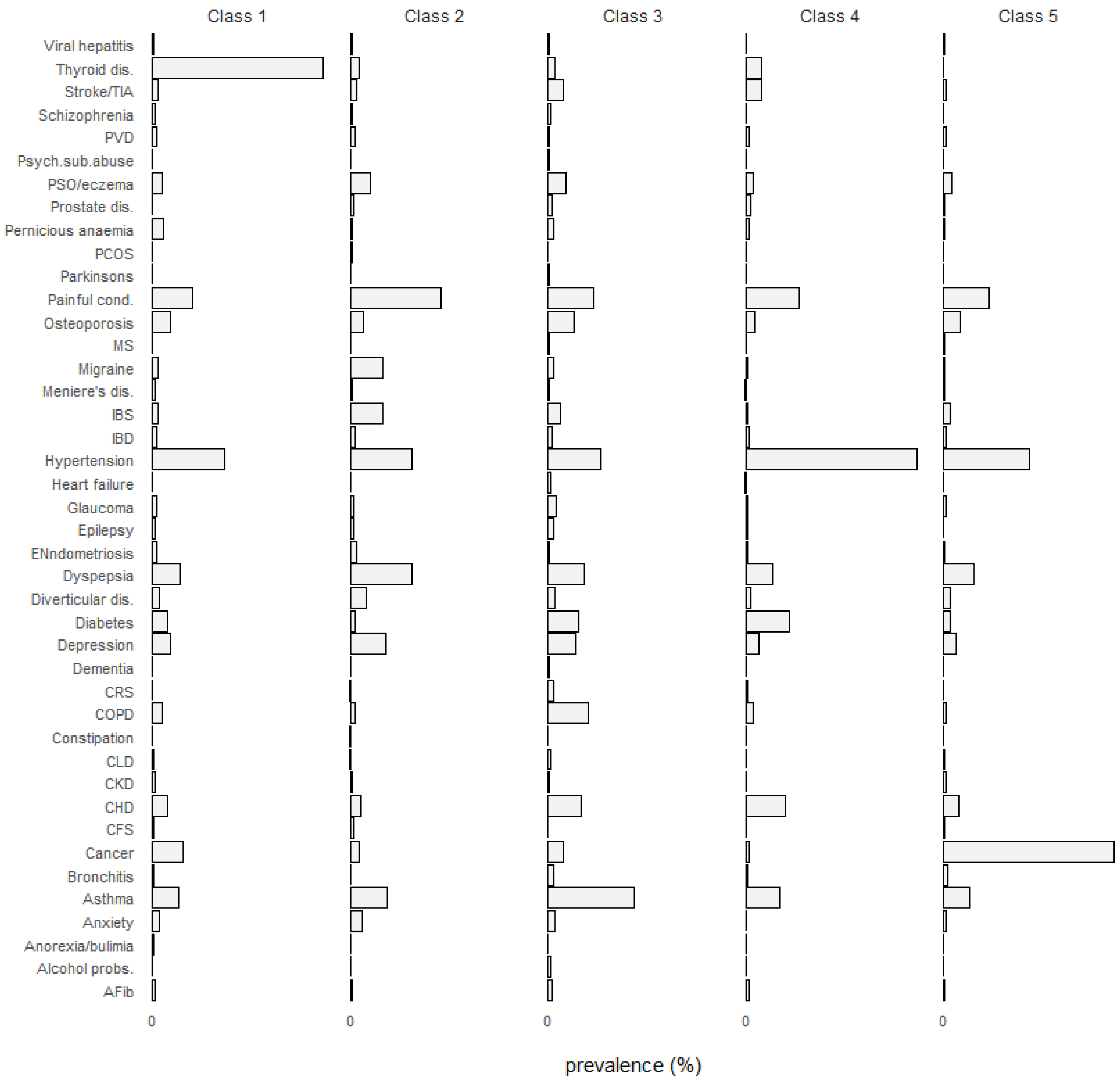

Figure S4 Latent class analysis of 42 long-term conditions (LTCs). 5-class solution showing within class prevalence of each condition. AFib-arterial fibrillation, CFS-chronic fatigue syndrome, CHD-coronary heart disease, CKD-chronic kidney disease, CLD-chronic liver disease, COPD-chronic obstructive pulmonary disease, CRS-chronic sinusitis, Diverticular .dis.-diverticular disease, IBD-inflammatory bowel disease, IBS-irritable bowel syndrome, Meniere's dis.-Meniere's disease, MS-multiple sclerosis, Painful cond.-painful conditions, PCOS-polycystic ovary syndrome, Prostate dis.-prostate disorders, PSO/eczema-psoriasis or eczema, Psych.sub.abuse-psychoactive substance abuse, PVD-peripheral vascular disease, Stroke/TIA-stroke or transient ischaemic attack, Thyroid dis.-thyroid disorders.

Figures S5 to S9 – Illustrate the 50 most prevalent long-term condition (LTC) combinations in each latent class. Black dots and lines indicate specific combination of LTCs. The top bar-chart indicates the number of participants who reported each combination of LTCs. The side bar-chart indicates the number of participants who reported each LTC.

AFib-arterial fibrillation, CFS-chronic fatigue syndrome, CHD-coronary heart disease, CKD-chronic kidney disease, CLD-chronic liver disease, COPD-chronic obstructive pulmonary disease, CRS-chronic sinusitis, Diverticular_dis.-diverticular disease, IBD-inflammatory bowel disease, IBS-irritable bowel syndrome, Meniere_dis.-Meniere's disease, MS-multiple sclerosis, Painful_conds-painful conditions, PCOS-polycystic ovary syndrome, PSO_eczema-psoriasis or eczema, Psychoactive-psychoactive substance abuse, PVD-peripheral vascular disease, Stroke_TIA-stroke or transient ischaemic attack, Thyroid_dis.-thyroid disorders.

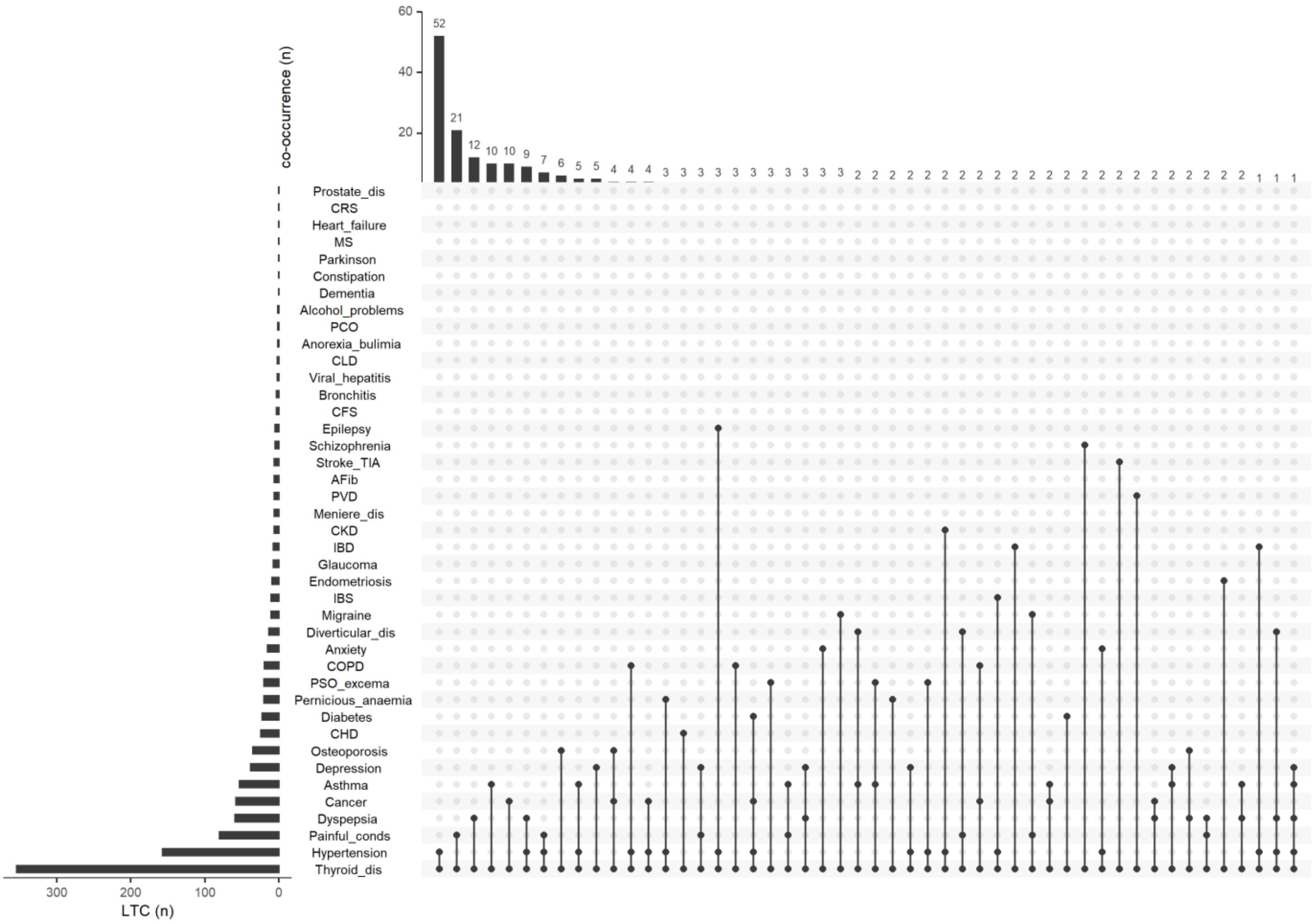

Figure S5 Class 1 Most prevalent long-tern condition (LTC) combinations (first 50 shown out of a total 173)

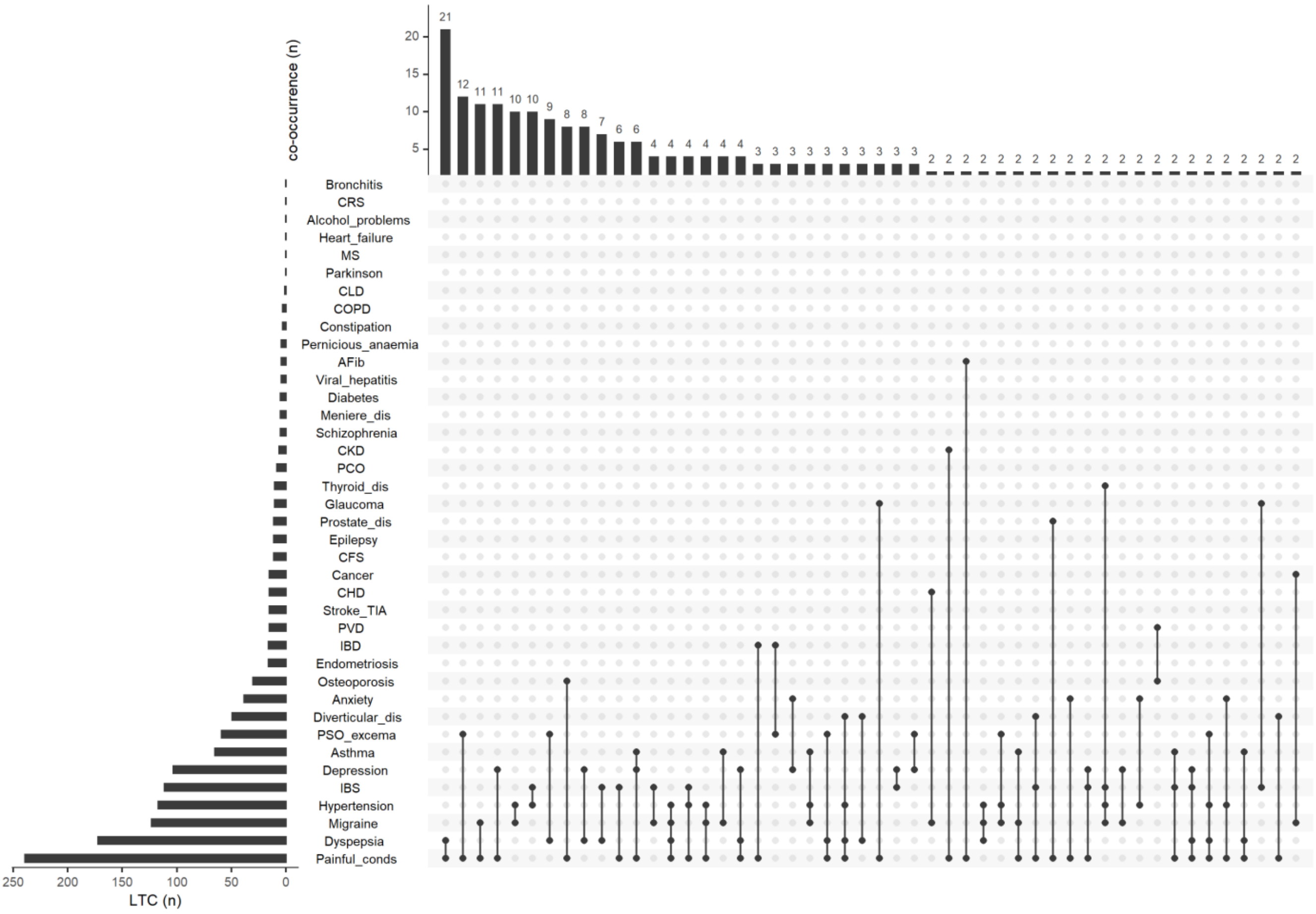

Figure S6 Class 2 Most prevalent long-term condition (LTC) combinations (first 50 shown out of a total of 257)

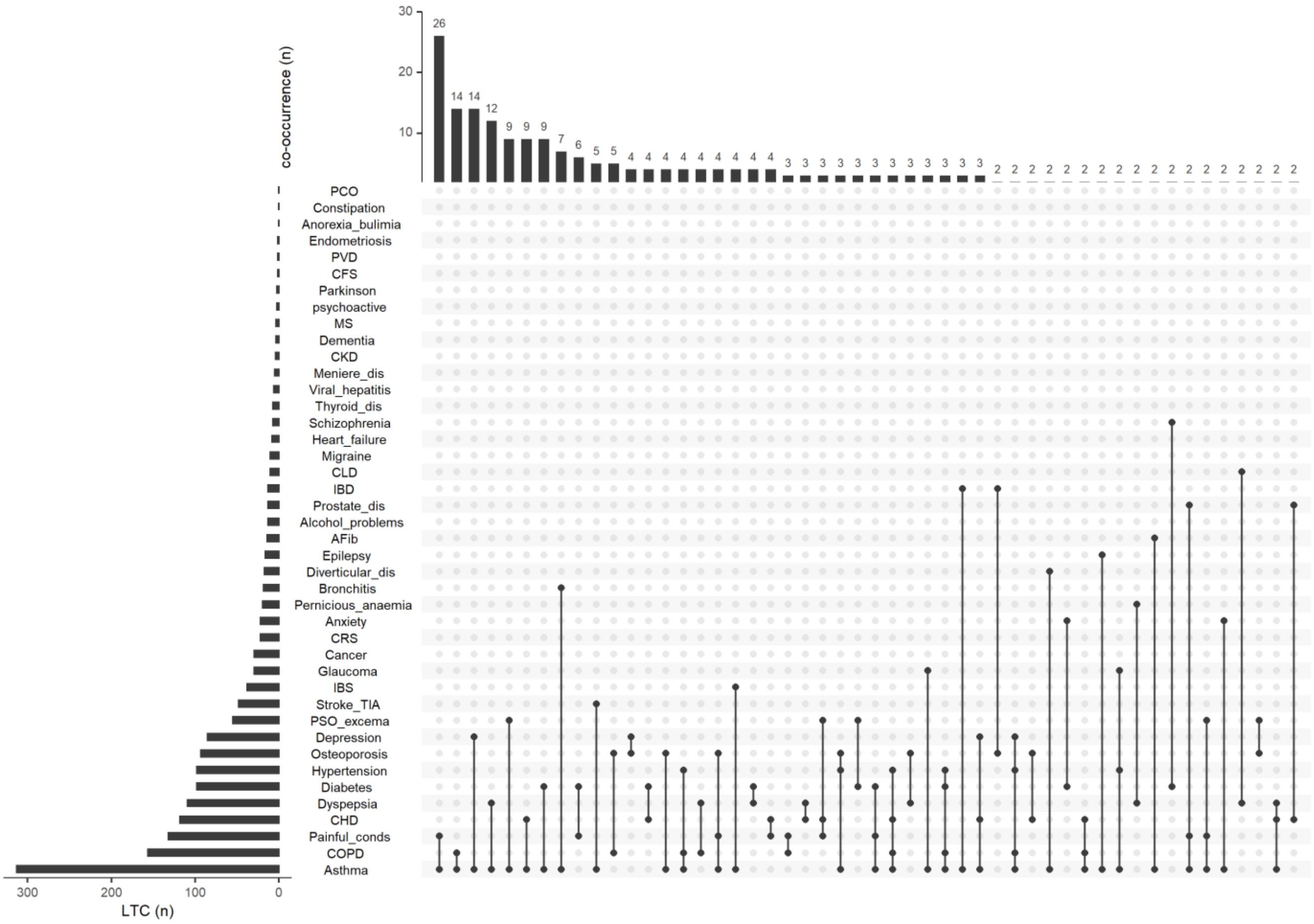

Figure S7 Class 3 Most prevalent long-term condition (LTC) combinations (first 50 shown out of a total of 334)

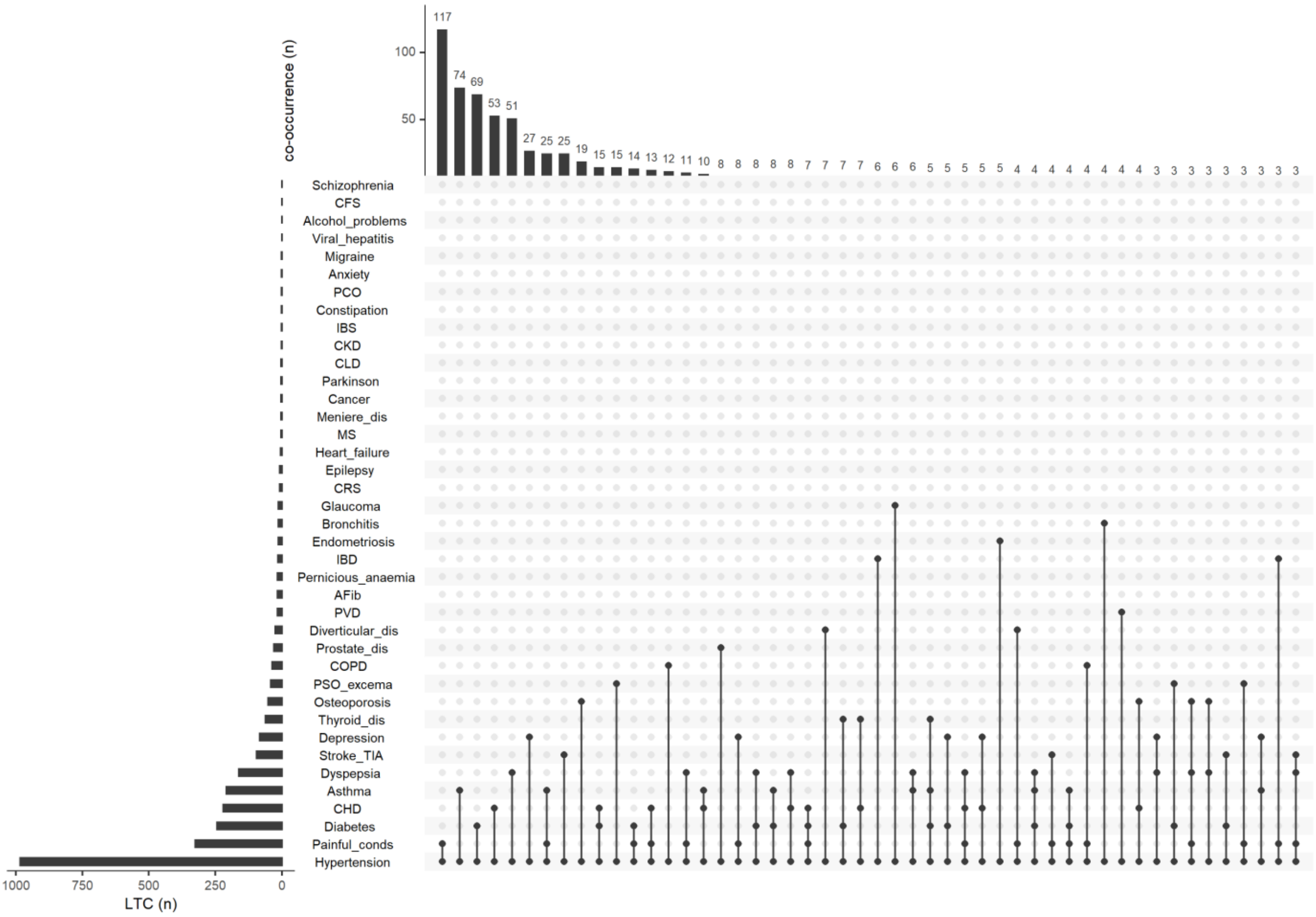

Figure S8 Class 4 Most prevalent long-term condition (LTC) combinations (first 50 shown out of a total of 266)

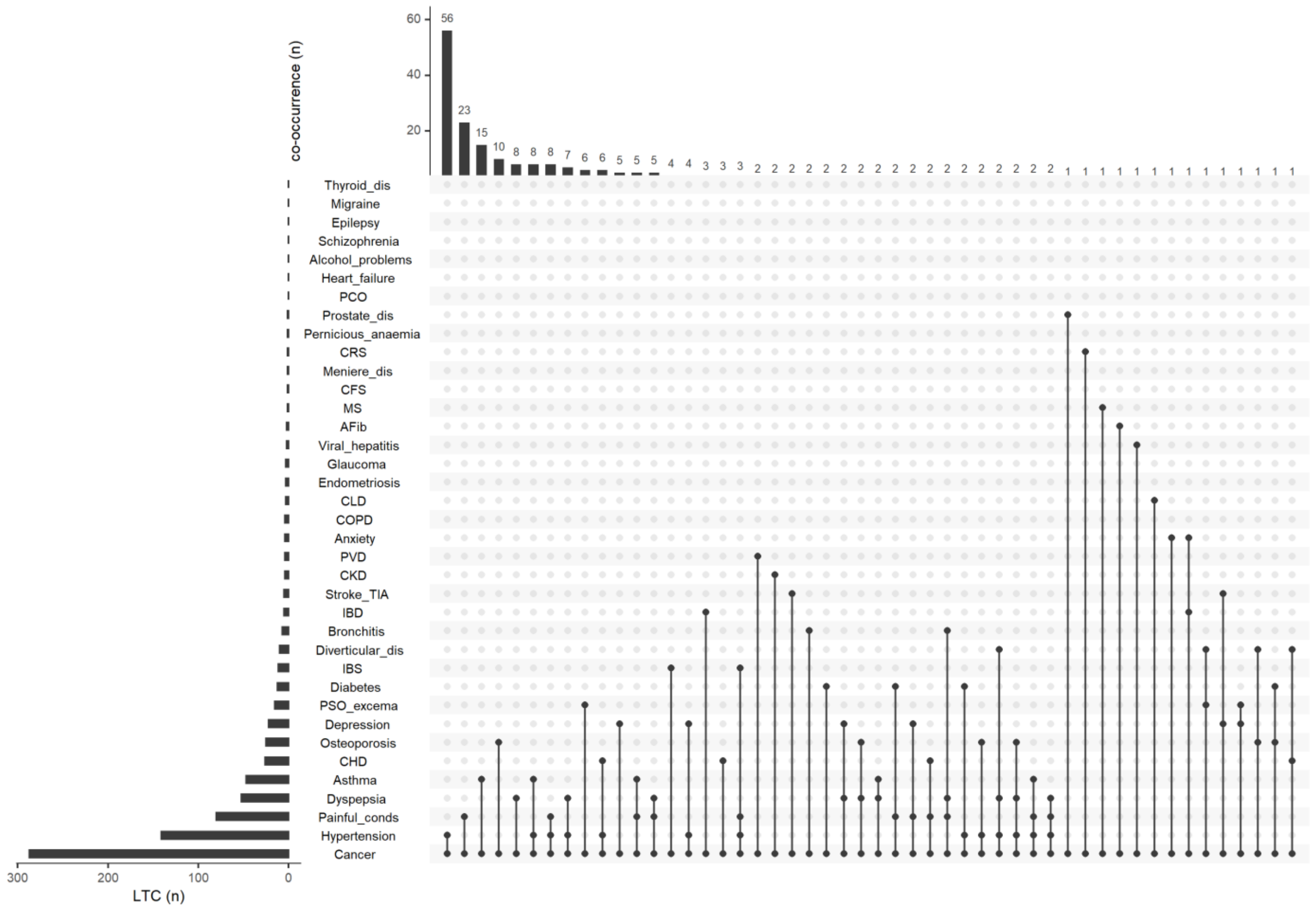

Figure S9 Class 5 Most prevalent long-term condition (LTC) combinations (first 50 shown out of a total of 108)
